# Neutrophil-to-lymphocyte ratio as prognostic indicator in gastrointestinal cancers: a systematic review and meta-analysis

**DOI:** 10.18632/oncotarget.16291

**Published:** 2017-03-16

**Authors:** Randy C. Bowen, Nancy Ann B. Little, Joshua R. Harmer, Junjie Ma, Luke G. Mirabelli, Kyle D. Roller, Andrew Mackay Breivik, Emily Signor, Alec B. Miller, Hung T. Khong

**Affiliations:** ^1^ Department of Oncology, University of Utah, Salt Lake City, Utah, USA; ^2^ Department of Pharmacotherapy, University of Utah, Salt Lake City, Utah, USA

**Keywords:** neutrophil-to-lymphocyte ratio, gastrointestinal cancers, prognostic indicator, overall survival, biomarkers

## Abstract

An accurate, time efficient, and inexpensive prognostic indicator is needed to reduce cost and assist with clinical decision making for cancer management. The neutrophil-to-lymphocyte ratio (NLR), which is derived from common serum testing, has been explored in a variety of cancers. We sought to determine its prognostic value in gastrointestinal cancers and performed a meta-analysis of published studies using the Meta-analysis Of Observational Studies in Epidemiology guidelines. Included were randomized control trials and observational studies that analyzed humans with gastrointestinal cancers that included NLR and hazard ratios (HR) with overall survival (OS), disease-free survival (DFS), progression-free survival (PFS), and/or cancer-specific survival (CSS).

We analyzed 144 studies comprising 45,905 patients, two-thirds of which were published after 2014. The mean, median, and mode cutoffs for NLR reporting OS from multivariate models were 3.4, 3.0, 5.0 (±IQR 2.5-5.0), respectively. Overall, NLR greater than the cutoff was associated with a HR for OS of 1.63 (95% CI, 1.53-1.73; P < 0.001). This association was observed in all subgroups based on tumor site, stage, and geographic region. HR for elevated NLR for DFS, PFS, and CSS were 1.70 (95% CI, 1.52-1.91, *P* < 0.001), 1.64 (95% CI, 1.36-1.97, *P* < 0.001), and 1.83 (95% CI, 1.50-2.23, *P* < 0.001), respectively.

Available evidence suggests that NLR greater than the cutoff reduces OS, independent of geographic location, gastrointestinal cancer type, or stage of cancer. Furthermore, DFS, PFS, and CSS also have worse outcomes with elevated NLR.

## INTRODUCTION

As the genomic revolution advances, more molecular biomarkers have been discovered that can serve as druggable targets or prognosticators of therapeutic efficacy, disease recurrence, or survival. Though exciting to provide these novel options for patients, the cost of employing these molecular markers and the time to send off and obtain results can be significant. Therefore, even in this day of genomic and proteomic advancements, a simple, inexpensive and readily available prognostic marker is still highly desirable.

One of the simplest and most readily available tests in the clinic is the complete blood cell count (CBC), which reports the absolute neutrophil count (ANC) and absolute lymphocyte count (ALC). The neutrophil-to-lymphocyte ratio (NLR), calculated by dividing the ANC by the ALC, can serve as an index of systemic inflammatory response in critically ill patients [1]. The microenvironments of inflammation, created by mediators and cellular effectors, are recognized as both a condition that leads to cancer development, as well as an outcome that results from cancer cell growth [2]. Building on the six classic hallmarks of cancer, tumor-promoting inflammation is now widely accepted as an enabling characteristic, which supports multiple cancer hallmark capabilities [3]. Therefore, NLR may help to reflect systemic inflammation in patients with cancer and their immunologic capacity to mount an attack against the malignant cells. An increasing number of recent reports suggest that NLR can be used as a prognostic marker in various malignancies.

This meta-analysis aims to determine the prognostic value of NLR in gastrointestinal (GI) cancers for overall survival (OS), disease free survival (DFS), progression free survival (PFS), and cancer-specific survival (CSS).

## RESULTS

**Figure 1:**
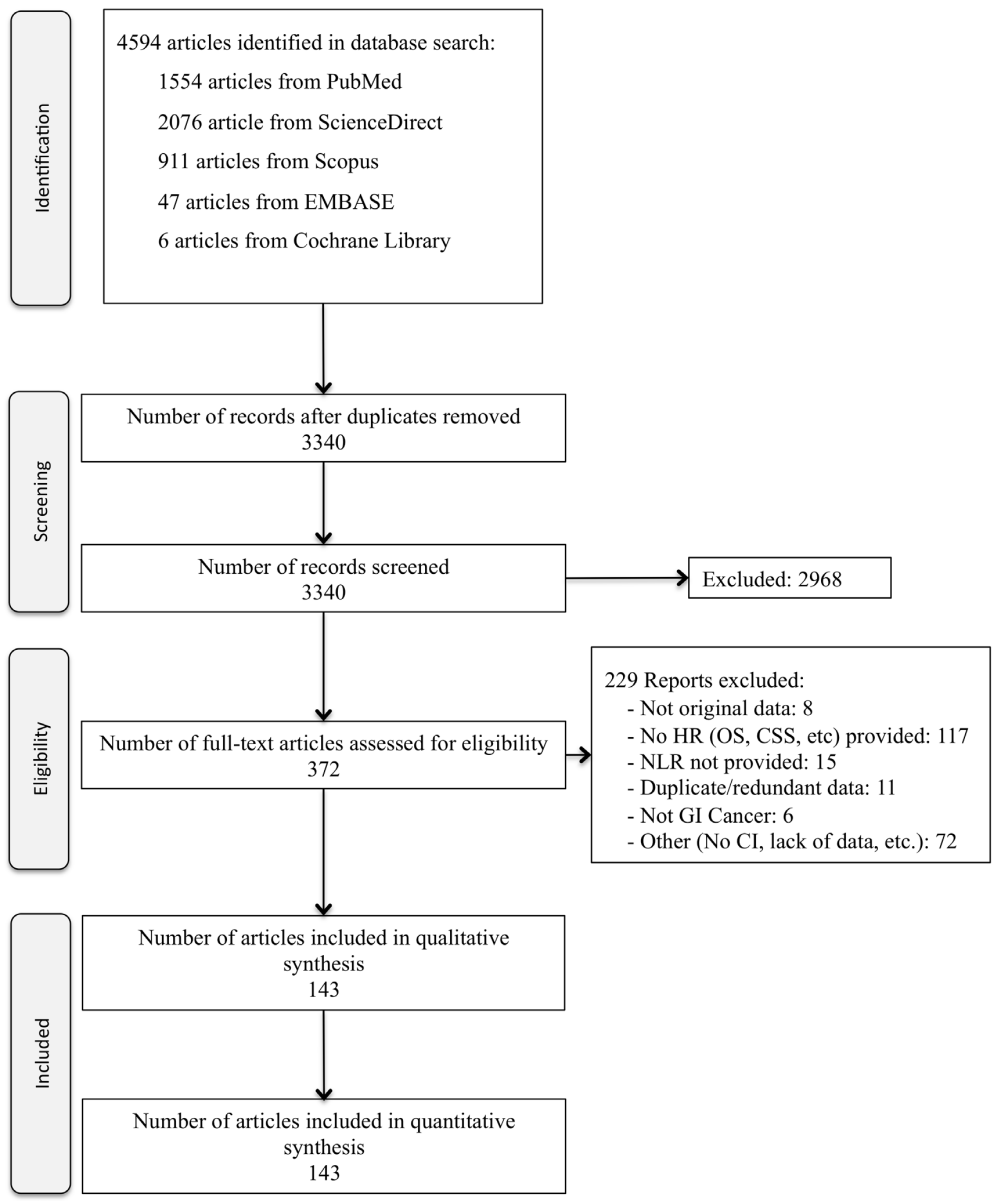
Flowchart of study selection Flow diagram of study selection process for the neutrophil-lymphocyte ratio meta-analysis of GI cancers. HR = Hazard Ratio; OS = Overall survival; CSS = Cancer specific survival; NLR = Neutrophil-to-Lymphocyte ratio; GI = Gastrointestinal; CI = Confidence Interval.

We identified 4,594 articles of which 372 were selected for full-text review (Figure 1). 143 articles met the inclusion criteria, of which 141 were retrospective cohort studies, one was a retrospective case control study, and one was a prospective RCT. One article included 2 cancer site studies [4], resulting in 144 cancer site studies. Publication dates ranged from 2008 to 2016 with more than two-thirds published after 2014. A total of 118, 53, 10, and 19 studies performed analyses on OS, DFS, PFS, and CSS, respectively. Gastrointestinal disease site included cholangiocarcinoma, colorectal carcinoma, esophageal carcinoma, gastric cancer, gastrointestinal stromal tumor, hepatocellular carcinoma, and pancreatic cancer. Of the 144 cancer site studies, 52 included non-metastatic cancer, 23 included metastatic cancers, and 69 included a mix of both disease stages. Participants in 71 studies received more than one treatment modality (chemotherapy, surgery, radiation), participants in 52 studies underwent only surgery as therapy, 19 studies reported only chemotherapy, and participants in two studies were treated only with radiation therapy. Studies were conducted in multiple countries including Australia (*N* = 4; 2.8%), Austria (*N* = 3; 2.1%), Canada (*N* = 3; 2.1%), China (*N* = 48; 33.3%), Ireland (*N* = 1; 0.7%), Italy (*N* = 4; 2.8%), Japan (*N* = 30; 20.8%), Singapore (*N* = 1; 0.7%), Korea (*N* = 21;14.6%), Taiwan (*N* = 5; 3.5%), Turkey (*N* = 2; 1.4%); United Kingdom (*N* = 13; 9%), and the United States (*N* = 9; 6.3%). The median cutoff for the NLR among all included studies was 3.0 (±IQR = 2.495-5.0). There were 229 studies excluded, most commonly due to the absence of primary outcome measurements (OS, DFS, PFS, and CSS) or the HR for these primary outcomes. Full text papers could not be located for 2 studies. In cases where there were redundant study populations between multiple publications, we only used the most recent publication and excluded prior studies (Table 1).

**Table 1:** Characteristics of included studies


Characteristics			Studies		Patients		References
			N=144	(%)	N=45,905	(%)	
Year of Publication							
		2008	1	(1)	440	(1)	[41]
		2009	3	(2)	614	(1)	[42–44]
		2010	5	(3)	1,538	(3)	[45–49]
		2011	8	(6)	2,231	(5)	[50–57]
		2012	12	(8)	4,518	(10)	[4, 58–67]
		2013	14	(10)	3,992	(9)	[68–81]
		2014	32	(22)	8,260	(18)	[82–113]
		2015	48	(34)	17,501	(38)	[5, 114–160]
		2016	21	(15)	6,474	(14)	[161–181]
Study Type							
	Case control						
		Retrospective	1	(1)	93	(<1)	[62]
	Cohort						
		Retrospective	141	(99)	44,614	(98)	[4, 41–61, 63–181]
	Randomized control trial						
		Prospective	1	(1)	861	(2)	[5]
Hazard Ratios							
	Overall survival						
		Multivariate	110	(77)	36,884	(80)	[4, 5, 41–43, 47–61, 63–67, 69, 70, 72, 73, 77, 81, 84, 88, 91–104, 106, 107, 110–113, 115–119, 123–131, 134, 137–143, 146–148, 151–160, 164, 166, 168–171, 173–179, 181]
		Univariate	74	(51)	23,069	(50)	[41–45, 54, 55, 58, 59, 61, 64–66, 68, 69, 74, 77, 81, 82, 87, 91, 93, 94, 97–101, 105, 108, 110, 112–118, 121, 123–126, 128–130, 132, 134, 136, 137, 139, 140, 142, 144, 146–148, 150, 151, 155, 156, 158, 161, 166, 169, 171, 173–179, 181]
	Disease-free survival						
		Multivariate	44	(31)	14201	(31)	[4, 42, 46, 52, 55, 57, 58, 62, 66, 67, 70, 75, 76, 89, 90, 92, 96, 97, 99, 100, 106, 109, 110, 112, 116, 117, 119, 120, 122, 125, 139, 145, 149, 151, 156, 158, 165, 170, 171, 175, 176, 178, 179, 181]
		Univariate	35	(24)	7745	(17)	[42, 44, 46, 55, 58, 66, 74, 75, 78, 83, 89, 90, 97, 100, 110, 112, 116, 117, 122, 125, 135, 137, 139, 144, 145, 151, 155, 156, 158, 166, 171, 175, 178, 179, 181]
	Progression-free survival						
		Multivariate	7	(5)	1,203	(3)	[50, 61, 84, 102, 111, 118, 126]
		Univariate	6	(4)	615	(1)	[60, 61, 118, 126, 129, 147]
	Cancer-specific survival						
		Multivariate	15	(10)	4,586	(10)	[71, 79, 80, 89, 90, 103, 110, 119, 120, 133, 138, 145, 149, 173, 180]
		Univariate	13	(9)	3,400	(7)	[71, 79, 80, 85, 86, 89, 90, 110, 133, 145, 167, 172, 173]
Disease Site							
		Cholangiocarcinoma	4	(3)	1,272	(3)	[94, 105, 174, 175]
		Colorectal carcinoma	47	(32)	14,891	(32)	[4, 41, 43, 44, 46, 47, 50, 52, 58, 61, 63, 67, 68, 71, 75, 79, 85, 86, 89, 90, 97–100, 103, 110, 115, 117, 118, 122, 124–126, 135, 137, 138, 140, 145, 147–150, 155, 169, 172, 179]
		Esophageal carcinoma	14	(10)	4,101	(9)	[54, 55, 69, 111, 112, 120, 129, 133, 146, 167, 171, 173, 180, 181]
		Gastric cancer	23	(16)	11,196	(24)	[48, 53, 60, 62, 66, 72, 73, 84, 87, 88, 95, 104, 119, 127, 130, 131, 142, 159, 164]
		Gastrointestinal stromal tumor	4	(3)	630	(1)	[78, 83, 144, 165]
		Hepatocellular carcinoma	34	(24)	9,170	(20)	[42, 51, 56, 59, 64, 70, 74, 76, 77, 82, 91, 92, 96, 102, 106, 107, 109, 113, 116, 121, 123, 132, 136, 139, 152, 154, 156–158, 160, 168, 170, 176]
		Pancreatic cancer	18	(13)	4,642	(10)	[5, 45, 65, 80, 81, 93, 108, 114, 128, 134, 141, 151, 161–163, 166, 177, 178]
Disease Stage							
		Nonmetastatic	52	(36)	19,373	(41)	[4, 42, 46, 58, 59, 62, 68–70, 74, 76, 78, 83, 86, 88, 90, 96, 100, 106, 107, 109, 111, 114, 117, 120–122, 129–133, 135, 137, 140, 146, 149, 158, 159, 162, 165, 166, 168, 170, 171, 176, 178–181]
		Metastatic	23	(16)	5,343	(12)	[5, 41, 44, 50, 60, 61, 67, 82, 84, 95, 97, 101, 104, 115, 118, 124–126, 138, 147, 150, 172, 177]
		Mixed	69	(48)	21,189	(46)	[43, 45, 47–49, 51, 53–57, 63–66, 71–73, 75, 77, 79–81, 85, 87, 89, 91–94, 98, 99, 102, 103, 105, 108, 110, 112, 113, 116, 119, 123, 127, 128, 134, 136, 139, 141–145, 148, 151–157, 160, 161, 163, 164, 167, 169, 173–175]
Treatment Method							
		Resection	52	(36)	19,798	(43)	[42, 46, 48, 52, 62, 69, 70, 72, 74–76, 78, 79, 83, 85, 86, 92, 96, 104–106, 109, 116, 117, 119, 120, 122, 127, 130–133, 135, 139, 140, 142, 146, 149, 153, 157, 158, 163, 165–171, 175, 176, 178]
		Chemotherapy	19	(13)	4,071	(9)	[5, 45, 50, 51, 60, 61, 81, 84, 91, 102, 107, 108, 118, 121, 126, 134, 141, 147, 174]
		Radiation	2	(1)	366	(1)	[59, 161]
		Mixed	71	(49)	21,670	(47)	[4, 41, 43, 44, 47, 49, 53–58, 63, 64, 66–68, 71, 73, 77, 80, 82, 87–90, 93–95, 97–101, 103, 110–115, 123–125, 128, 129, 136–138, 143–145, 148, 150–152, 154–156, 159, 160, 162, 164, 172, 173, 177, 179–181]
NLR Cutoff							
		< 3.0	55	(38)	19,656	(43)	[47, 53, 57, 59, 62, 70, 72, 77–79, 83, 88, 90–92, 97, 98, 100–102, 105, 107, 111, 114, 116, 117, 119, 120, 123, 124, 126, 128–133, 140–142, 144–146, 148, 153, 156, 160, 162, 164, 169, 170, 173, 176, 180, 181]
		3.0 to 3.99	35	(24)	12,197	(27)	[4, 5, 51, 57, 60, 69, 73, 81, 84, 85, 87, 89, 94, 95, 104, 109, 110, 121, 122, 134–136, 138, 143, 147, 149, 155, 159, 163, 166–168, 175, 177]
		4.0 to 4.99	14	(10)	3,501	(8)	[46, 48, 54, 61, 68, 76, 82, 103, 106, 125, 137, 154, 157, 158]
		≥ 5.0	40	(28)	10,214	(22)	[41, 43–45, 49, 50, 52, 55, 58, 63–67, 71, 74, 75, 80, 86, 93, 96, 99, 108, 112, 113, 115, 118, 127, 139, 150–152, 165, 171, 172, 174, 178, 179]
Study Origin							
	Asia/Oceania						
		Australia	4	(3)	1,552	(3)	[5, 50, 93, 179]
		China	48	(33)	16,051	(37)	[45–47, 51, 56, 57, 65–67, 69, 70, 88, 91, 92, 100, 104–107, 110, 112–114, 116, 119–121, 123, 129, 132–134, 141–143, 146, 153, 156–160, 168, 170, 175, 176, 180, 181]
		Japan	30	(21)	7,704	(17)	[48, 49, 54, 61, 76, 79, 82, 87, 90, 95, 101, 102, 108, 109, 128, 135, 137, 139, 147, 148, 154, 155, 162, 163, 166, 167, 169, 172, 173, 178]
		Korea	21	(15)	6,880	(15)	[53, 60, 62, 63, 72, 73, 77, 84, 85, 89, 96, 99, 111, 130, 131, 145, 149, 150, 171, 174, 177]
		Singapore	1	(1)	300	(1)	[165]
		Taiwan	5	(4)	5,311	(12)	[4, 52, 59, 127]
	Europe/ Mediterranean						
		Austria	3	(2)	1,552	(2)	[68, 80, 124]
		Ireland	1	(1)	85	(0)	[81]
		Italy	4	(3)	699	(2)	[118, 122, 126, 164]
		Turkey	2	(1)	348	(1)	[83, 98]
		United Kingdom	13	(9)	2,689	(6)	[41, 42, 44, 58, 64, 71, 75, 86, 97, 103, 125, 138, 140]
	Northern America						
		Canada	3	(2)	1,506	(3)	[94, 117, 144]
		United States	9	(6)	1,968	(4)	[43, 55, 74, 78, 115, 136, 151, 152, 161]

### Overall survival

**Figure 2:**
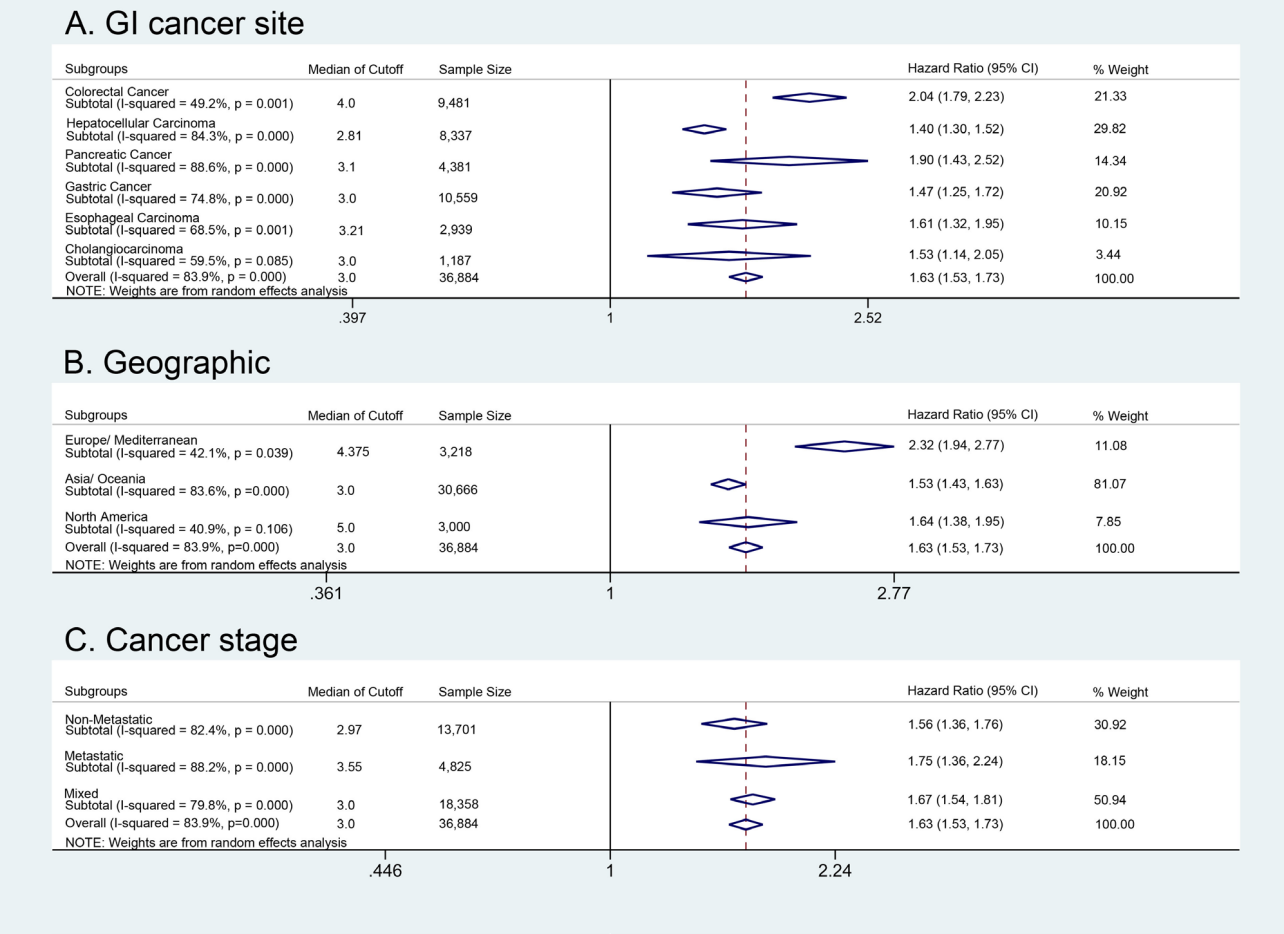
Overall survival analysis Overall survival analysis of NLR cut off, geographic location, and disease stage. **A**. Overall survival in patients with GI cancers and per individual GI cancer types. **B**. Overall survival analysis within geographic regions. **C**. Overall survival within each disease stage.

110 studies including 36,884 patients reported a HR for OS based on a multivariate model. 6 studies reported a HR for OS, but failed to use a multivariate model and therefore were not included in our main analysis. For an analysis of hazard ratios for OS using univariate models, see Table 2. The mean, median, and mode cutoff for NLR reporting OS from multivariate model was 3.4, 3.0, 5.0 (±IQR 2.5, 5.0), respectively with 39 studies using 5.0 compared to 25 studies using 3.0. A forest plot depicting OS for included studies is shown in Figure 2. Overall, NLR greater than the cutoff value was associated with a hazard ratio for OS of 1.63 (95% CI 1.53 to 1.73; *P* < 0.001).

**Table 2:** Sensitivity analysis

Studies	HR	UL of 95% CI	LL of 95% CI	P of heterogeneity chi-squared
Overall survival				
Model type				0.001
Multivariate	1.63	1.53	1.73	
Univariate	1.92	1.78	2.08	
C-index				0.002
Yes	1.45	1.35	1.56	
No	1.79	1.60	2.00	
NLR cutoff				0.026
≤3	1.54	1.43	1.64	
>3	1.83	1.60	2.10	
Stage				0.565
Metastatic	1.75	1.36	2.24	
Mixed	1.67	1.54	1.81	
Nonmetastatic	1.56	1.39	1.76	
Disease free survival				
Model type				0.142
Multivariate	1.71	1.52	1.91	
Univariate	1.99	1.69	2.36	
C-index				0.005
Yes	1.48	1.31	1.67	
No	2.10	1.70	2.60	
NLR cutoff				0.012
≤3	1.48	1.34	1.63	
>3	2.12	1.63	2.76	
Stage				0.799
Metastatic	1.51	0.90	2.53	
Mixed	1.78	1.42	2.22	
Nonmetastatic	1.66	1.45	1.91	
Progression free survival				
Model type				0.728
Multivariate	1.64	1.36	1.97	
Univariate	1.45	0.74	2.82	
C-index				0.785
Yes	1.76	1.28	2.41	
No	1.66	1.26	2.19	
NLR cutoff				0.140
≤3	1.51	1.28	1.78	
>3	2.27	1.36	3.81	
Stage				0.386
Metastatic	1.78	1.36	2.34	
Mixed	1.36	1.01	1.84	
Nonmetastatic	1.80	1.05	3.08	
Cancer specific survival				
Model type				0.117
Multivariate	1.83	1.50	2.23	
Univariate	2.27	1.89	2.72	
C-index				0.105
Yes	1.61	1.24	2.10	
No	2.12	1.73	2.60	
NLR cutoff				0.173
≤3	1.59	1.35	1.88	
>3	2.20	1.42	3.40	
Stage				0.049
Metastatic	1.93	1.40	2.66	
Mixed	2.18	1.68	2.84	
Nonmetastatic	1.37	1.04	1.80	

Abbreviations: HR = Hazard ratio, NLR = Neutrophil-to-lymphocyte ratio, C-index = Receiver operating characteristic curves for selection of cutoff,

The prognostic effect of NLR on OS among subgroups based on disease site is shown in Figure 2A. The disease site with the greatest HR for OS was colorectal cancer (HR = 2.04; 95% CI 1.79 to 2.33), followed by pancreatic cancer (HR = 1.90; 95% CI 1.43 to 2.52), esophageal carcinoma (HR = 1.61; 95% CI 1.32 to 1.95), cholangiocarcinoma (HR = 1.53; 95% CI 1.14 to 2.05), gastric cancer (HR = 1.47; 95% CI 1.25 to 1.72) and hepatocellular carcinoma (HR = 1.40; 95% CI 1.30 to 1.52).

The prognostic effect of NLR on OS among subgroups based on geographic location is shown in Figure 2B. The largest portion of patients in the OS analysis was in the Asia/Oceania region (81.07%), but they had the lowest HR at 1.53 (95% CI 1.43 to 1.63). By comparison, the HR for Northern America was 1.64 (95% CI 1.38 to 1.95) and the HR for Europe/Mediterranean was 2.32 (95% CI 1.93 to 2.77).

The prognostic effect of NLR on OS among subgroups based on disease stage is shown in Figure 2C. The median NLR cutoff value for metastatic disease was 3.55 (±IQR 3 to 5), whereas the median NLR cutoff value for nonmetastatic disease was 2.97 (±IQR 2 to 4). This difference between the NLR cutoff values in metastatic versus nonmetastatic disease was statistically significant (*P* = 0.0134). The HR for a high NLR on OS in metastatic disease was 1.75 (95% CI 1.36 to 2.24). The HR for nonmetastatic disease was 1.56 (95% CI 1.36 to 1.76). The HR for mixed disease stages was 1.67 (95% CI 1.54 to 1.81). There was no statistical difference in OS between the nonmetastatic and metastatic cancer groups at their NLR cutoff values (0.64; d.f. = 1; *P* = 0.423). Sensitivity analyses of the included studies are reported in Table 2. In analyzing OS, we found statistically significant differences in HR when comparing multivariate and univariate analyses (*P* = 0.001), presence of C-index to select NLR cutoff (*P* = 0.002), and NLR < 3 compared to > 3 (*P* = 0.026). However, there was no difference when comparing disease stages (metastatic, mixed, non-metastatic (*P* = 0.565)). The meta-regression scatter plot showed minor but statistically significant association between NLR cutoff and the hazard ratio for OS (β = 0.224; *P* = 0.019) (Supplementary Figure 1).

### Disease-free (recurrence-free) survival

**Figure 3:**
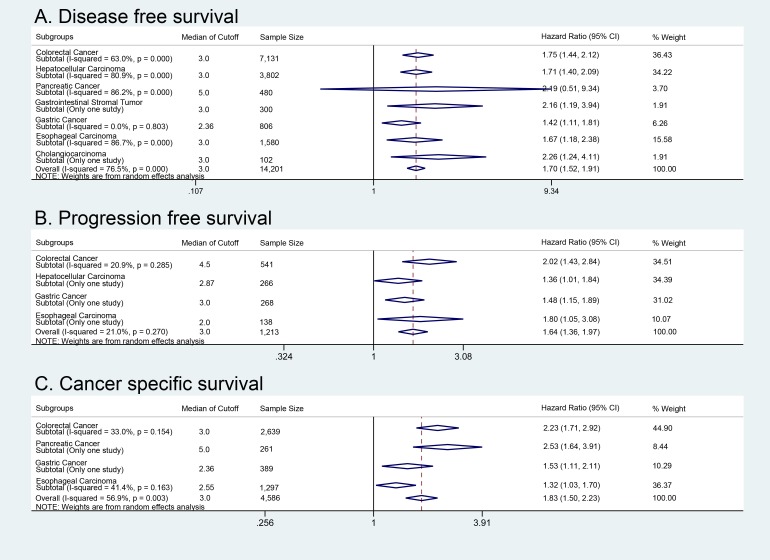
Disease and progression free survival with cancer-specific survival analysis Subgroup survival analysis based on cancer site. **A**. Disease-free survival of five cancer types. **B**. Progression free survival of four cancer types. **C**. Cancer specific survival of four cancer types.

44 studies including 14,201 patients reported hazard ratios for DFS or RFS analyzed in a multivariate model. The median cutoff for a high NLR was 3 (±IQR 2.4 to 5.0). For DFS, NLR greater than the cutoff value was associated with a HR of 1.70 (95% CI 1.52 to 1.91, *P* < 0.001; I-squared = 76.5%). There were not statistically significant differences among the hazard ratios of different subgroups based on disease site (Heterogeneity chi-squared = 3.74 (d.f. = 6) *P* = 0.711) (Figure 3A). For an analysis of hazard ratios for DFS using univariate models, see Table 2.

### Progression-free survival

7 studies including 1,213 patients reported hazard ratios for PFS analyzed in a multivariate model. The median cutoff for a high NLR was 3 (±IQR 2 - 5). For PFS, NLR greater than the cutoff value was associated with a HR of 1.64 (95% CI 1.36 to 1.97, *P* < 0.001; I-squared = 21.0%). There were not statistically significant differences among the hazard ratios of different subgroups based on disease site (Heterogeneity chi-squared = 3.37 (d.f. = 3) *P* = 0.338) (Figure 3B). For an analysis of hazard ratios for PFS using univariate models, see Table 2.

### Cancer-specific survival

15 studies including 4,586 patients reported hazard ratios for CSS analyzed in a multivariate model. The median cutoff for a high NLR was 3 (±IQR 2.36 - 4.75). For CSS, NLR greater than the cutoff value was associated with a HR of 1.83 (95% CI = 1.50 to 2.23, *P* < 0.001; I-squared = 56.9%). There was a small, but statistically significant difference in the hazard ratios for CSS between the colorectal cancer and esophageal carcinoma subgroups (Heterogeneity chi-squared = 7.82; (d.f. = 1); *P* = 0.005) (Figure 3C). For an analysis of hazard ratios for CSS using univariate models, see Table 2.

The heterogeneity observed in these analyses was largely due to observational studies and variation between comparable characteristic, such as the use of C-index to determine the NLR cut-off value and whether the patient had metastatic versus non-metastatic lesions (Table 2). We found that the HR for studies without C-index justification was 1.79 (95% 1.60 to 2.00), and the HR for studies using C-index as justification is 1.45 (95% 1.35 to 1.56). The p-value for chi-squared test is 0.002 for these two groups. This indicated that studies which failed to report how they chose cutoff are more likely to report higher HR, compared to studies reported how they chose the cutoff of NLR.

### Risk of bias summary

Confounding was the most severe risk of bias within our included studies (Table 3 and Supplementary Figure 2). The retrospective nature of most studies accounted for the high likelihood for confounding variables. Many studies performed analyses to adjust for confounders. Bias in selection of participants and in measurement of interventions was low risk in the majority of studies. In terms of bias due to departures from intended interventions, three studies displayed moderate risk and one had serious risk. Bias due to missing data was primarily low risk due to the lack of missing observations and incomplete data reporting. Bias in selection of the reported outcomes was mostly attributed to lack of measurement reporting and lack of utilizing numerous analysis methods. A funnel plot revealed potential publication bias as seen with asymmetric distribution (Supplementary Figure 3). The Egger's test indicated possible publication bias with the regression line not originating in the Y-axis zero (Bias = 4.165 (95% CI 3.546 to 4.784) *P* < 0.001) as well as a *P* < 0.05 indicating small study effect (Supplementary Figure 4).

**Table 3:** Risk of bias percent summary

Risk of Bias Summary Analysis							
Risk of Bias Severity	Bias due to confounding	Bias in selection of participants into study	Bias in measurement of interventions	Bias due to departures from intended interventions	Bias due to missing data	Bias in measurement of outcomes	Bias in selection of reported result
Low	N=8; 6%	N=136; 95%	N=136; 95%	N=139; 97%	N=135; 94%	N=143; 100%	N=127; 89%
Moderate	N=112; 78%	N=7; 5%	N=7; 5%	N=3; 2%	N=8; 6%	N=0; 0%	N=16; 11%
Serious	N=21; 15%	N=0; 0%	N=0; 0%	N=1; 1%	N=0; 0%	N=0; 0%	N=0; 0%
Critical	N=2; 1%	N=0; 0%	N=0; 0%	N=0; 0%	N=0; 0%	N=0; 0%	N=0; 0%

## DISCUSSION

An accurate, time efficient, and inexpensive prognostic indicator is needed to reduce cost and assist with clinical decision making for cancer management. In this systematic review and meta-analysis, we identified over a decade and a half of data from 45,905 GI cancer patients that underwent neutrophil-to-lymphocyte ratio testing to determine OS as well as DFS, PFS, or CSS. 143 studies were retrospective, with one correlation study of NLR with a phase III RCT [5]. We report overall moderate risk of bias in our studies due the effects of retrospective analyses with confounding, small sample-size, and variation in some comparable measurements. Colorectal, gastric, and hepatocelluar were the most common cancer sites. Asian populations were the most frequently studied, representing 76% of the total number of patients. The pooled HR for OS was 1.63 (95% CI 1.53 to 1.73), indicating that patients with NLR higher than cutoff were 63% more likely to die than patients with NLR lower than the cutoff. OS greater than NLR cutoff was independent of geographic location, GI cancer type, or stage of cancer (Figure 2). The overall median NLR cutoff for OS was 3.0, which was lower than other studies comparing solid tumors [6]. Our analysis shows that a NLR greater than the cutoff value predict worse OS.

Overall HR of DFS (1.70), PFS (1.64), and CSS (1.83), also suggest that elevated NLR indicate worse prognosis. However, since PFS analyzes disease progression, a low NLR cutoff may not result in a significant difference; consequently, a higher cutoff level may be more appropriate for metastatic disease.

Across all geographic locations we found that patients with elevated NLR were more likely to have worse OS. This further supports the prognostic value of NLR in GI cancers, independent of genetic variation at the population level. Individual-level analyses are needed to further validate this hypothesis. Interestingly, studies of European/Mediterranean patients had a significantly larger HR than studies from Asia/Oceania and North America. This could mean that people of Europe/Mediterranean are more sensitive to high NLR for worse OS, or that Europe/Mediterranean studies are more likely to report a higher HR. Possible confounding factors include poor study design, difference in patterns of care in geographic locations or small sample sizes. Despite these differences, our geographic analysis supports NLR above cutoff as a predictive index for overall survival.

The heterogeneity observed in these analyses was largely due to observational studies and variation between comparable characteristic, such as the use of C-index to determine the NLR cut-off value and whether the patient had metastatic versus non-metastatic lesions (Table 2). We found that the HR for studies without C-index justification was 1.79 (95% 1.60 to 2.00), and the HR for studies using C-index as justification is 1.45 (95% 1.35 to 1.56). The p-value for the chi-squared test was 0.002 for these two groups. This suggests that studies without C-index justification were more likely to report higher HR.

### Prognostic value of the NLR

The association between chronic inflammation and carcinogenesis has long been recognized, with examples such as gastric reflux and esophageal adenocarcinoma, *H. pylori* infection and gastric cancers, and chronic colonic ulcers and adenocarcinoma of the colon. More recently, the role of inflammation in tumor progression has been explored, with particular focus on the tumor microenvironment. Interactions between tumor and immune cells result in manipulation and misregulation of the immune response. Elevated NLR suggests a systemic inflammatory state and can be indicative of neutrophilia, lymphopenia, or a combination of both. It has been linked to disease states including but not limited to endometriosis [7], acute coronary syndrome [8], Alzheimer's disease [9], and a variety of cancers. Subsequent investigations have explored its use as a prognostic factor.

Neutrophilia can occur in cancer patients due to increased myeloid cell production, potentially resulting from ectopic colony-stimulating factor [10, 11]. Not only can circulating neutrophil levels rise, but neutrophils can localize to the tumor due to multiple factors, including general inflammatory signals like IL-1 and TNF-alpha [12], as well as IL-8 release triggered by hypoxic conditions of the tumor microenvironment [13]. Once present, TGF-β [14] produced by altered stromal cells [15] can activate neutrophils. Although many of the details are unclear, neutrophils are thought to primarily propagate a pro-tumor environment by secreting molecules such as VEGF, MMP-9 [16], and reactive oxygen species [17]. The respective effects of these molecules include promoting angiogenesis and tumor growth, degrading the ECM and providing favorable conditions for metastasis, and potentiating genome instability and tumor evolution. A recent study also demonstrated the expression of the T-cell-negative-regulatory molecule PD-L1 on tumor infiltrating neutrophils that could functionally inhibit the activation of T cells [18].

Lymphopenia in the context of cancer also suggests more aggressive disease progression. Examinations of the tumor microenvironment have shown a correlation between tumor-infiltrating lymphocytes (TILs) and prognosis in gastrointestinal [19, 20] and many other cancers [21], with decreased TIL populations linked to worse survival, and vice versa. The details, however, of lymphopenia are more convoluted than that of neutrophilia, in part due to the varied subsets and roles of lymphocytes. The immune system can produce tumor-specific CD8+ cytotoxic T cells (CTLs) that act to inhibit tumor progression [22], and thus lymphopenia suggests that the immune system is unable to perform anti-tumor activities. Tumors are postulated to evade the cytotoxic effects of CTLs [23] via evolving anti-apoptotic genes [24] or modulating the expression levels of cell-cell interaction proteins [25, 26]. CD4+ T cells are also found in the tumor microenvironment, the majority of which are regulatory T cells (Tregs) and Th17 cells [27]. Tregs are elevated in many cancers and are considered to be generally immunosuppressive, thus limiting the host immune response to the tumor and enabling tumor progression. While there is nuance when exploring the different lymphocyte subtypes and comparing tumors of different origins, in broad terms, lymphocytes have anti-tumor activity and thus lymphopenia indicates an environment conducive to tumor progression. Whether due to neutrophilia or lymphopenia, elevated NLR physiologically suggests an inability of the immune system to suppress cancer progression. Consequently, the NLR cut-off value leverages the balance of neutrophilia and leukopenia to suggests a quantitative index for overall survival in GI cancer patients.

NLR offers a noninvasive, low-cost, early opportunity to assess patient status and prognosis. Costs of cancer are increasing rapidly; in the US alone, an estimated $124.57 billion was spent in 2010 and that is projected to grow to $157.77 billion by 2020 [28]. These costs are concentrated in initial and terminal stages across multiple types of cancer, including but not limited to gastrointestinal cancers [29]. The majority of these expenses are incurred via hospital costs [30] and increases are largely driven by growing use and cost of chemotherapy and radiation therapy [31]. NLR could be used, for instance, prior to intervention to allow patients and physicians to make better decisions about the course of treatment that may be more cost-effective. For example, BRAF inhibitor has been shown to induce CD8+ T cells infiltration into tumors and may even be dependent on immune infiltration for its anticancer activity [32]. Therefore, NLR could potentially be used to select patients who may or may not benefit from BRAF inhibitor. Determining which patients will most benefit from treatment is especially important in light of increasing treatment costs [33]. NLR does not have the specificity of biomarkers derived from tumor tissue, such as analysis of gene mutations or protein expression levels, and hence has limitations on the applicability of some targeted therapies. However, many of these other biomarkers currently in use or development require obtaining tissue and are orders of magnitude more expensive than calculating NLR.

Elevated NLR is particularly attractive as a prognostic biomarker for cancer due to its affordability and accessibility. NLR is derived from complete blood count (CBC) analysis, which is regularly measured in cancer patients, and thus does not pose an additional cost or burden to patients or the medical system. Furthermore, the technology to analyze CBC, and thus NLR, is found beyond developed economies. Efficacious and inexpensive tools need to be explored for global use to deal with the rising incidence of cancer [34]. Developing and employing NLR as a prognostic biomarker for gastrointestinal cancers has the potential to impact a large number of patients and to improve clinical decision-making. Refining our understanding of NLR via continued and prospective study, as well as finding other low-cost biomarkers, should be a future focus in order for medical systems around the globe to improve health care access.

### Study strengths and limitations

We performed a detailed literature search using 5 search engines aggregating data from over 45,500 patients. We included both English and non-English publications. Furthermore, we used Cochrane and other risk of bias analysis tools to provide insight to the weaknesses and strengths of each study. The funnel plot asymmetry suggests publication bias, which has a variety of potential sources. First, extrapolating from the results of the sensitivity analysis (Table 2), NLR might have a better predictability for patients with an advanced cancer stage. We did not have an inclusion criteria for the cancer stage. Among the 112 studies which reported an adjusted HR for OS, 57 studies did not report the cancer stage of patients. Second, we did not limit the types of treatment and cancer in the pooled analysis. The NLR may have different predictability for patients receiving different treatments. Another possibility is the heterogeneity in the methodological quality of each study. Confounding effect was reported as overall moderate as a consequence of the retrospective analyses, and could contribute to funnel plot asymmetry. Limitations of this study include the predominance of observational studies and lack of RCTs. Further complication to the primary outcomes are the known correlation of NLR with other life threatening condition such as cardiovascular [35, 36], renal [37], and hepatic disease [38]. Therefore, to mitigate confounding, incorporating NLR in future RCTs is needed.

## CONCLUSIONS

Our meta-analysis pooled 45,905 patients to assess the prognostic indication of NLR with GI cancers reported in 144 studies of variable risk of bias. These studies evaluated the prognostic significance of elevated NLR in different GI cancers. Our meta-analysis suggests that across all GI cancers identified in this study, a NLR greater than cutoff values indicates reduced OS regardless of geographic location or cancer stage. Furthermore, worse DFS, PFS, and CSS outcomes were associated with high NLR. The individual cancer types analyzed and disease stages had varying median NLR cutoff values that appear to predict survival prognosis and could be used for appropriate treatment planning. NLR should be included in correlative studies in future clinical trials to further assess and validate these findings.

## MATERIALS AND METHODS

We conducted a meta-analysis of published literature using the Meta-analysis Of Observational Studies in Epidemiology (MOOSE) guidelines [39]. We considered studies that compared serum NLR to the OS, DFS, PFS or CSS of patients with GI cancers. The primary endpoints were overall survival (OS) by cancer site, geographic location, and cancer stage using multivariate analysis. We included DFS, PFS and CSS as secondary endpoints analyses based on cancer site. Univariate analyses of OS, DFS, PFS and CSS for each GI cancer type were also performed.

### Inclusion/exclusion criteria

We included studies that met the following criteria: 1) randomized controlled trials (RCT), quasi-RCT, cohort, or case control; 2) patient size greater than *N* = 20 patients; 3) analyzed humans with GI cancers that included NLR and hazard ratios with associated 95% confidence interval with OS, DFS, PFS, and CSS. We defined GI cancers as cancers that originated from structures between the esophagus and rectum, including the hepatobiliary-pancreatic system. We excluded studies that did not report original data, did not have HR or confidence interval for the survival analysis, lacked NLR, were duplicates, or presented redundant patient populations

### Database search

We identified published studies from PubMed (National Library of Medicine), ScienceDirect (Elsevier), Scopus (Elsevier), EMBASE (Embase.com), and Cochrane Library (Wiley Interscience) electronic databases from inception to March 3 2016. There were no language restrictions. We developed a detailed search strategy specific for each electronic database to enhance our search results (online Supplement). EndNote X7 was used for de-duplication (EndNote, Thomson Reuters).

### The review process/reviewer

Six reviewers independently assessed articles in 3 pairs (E.S., and A.M.B.; J.R.H., and N.A.B.L.; L.G.M and K.D.R.). The titles and abstracts (if available) were screened. Full-text copies for all potentially relevant articles were reviewed independently by at least 2 reviewers for inclusion and data collection. A third reviewer reconciled disagreements. Non-English articles had one reviewer, facilitated by language interpreters. For data extraction we used pre-made data entry sheets with details found in Supplementary Methods of the Supplement.

### Risk of bias

For the complete methods of our risk of bias analysis, see eMethods. In short, all papers were analyzed for bias using A Cochrane Risk of Bias Assessment Tool: For Non-Randomized Studies of Interventions (ACROBAT-NRSI) [40]. Additionally, random effect Begg's funnel plot and Egger's linear regression were created to evaluate publication bias.

### Statistical analysis

All analyses were conducted by using STATA statistical software, v 14 (Stata Corp LP, College Station, TX, USA). Hazard ratios and corresponding 95% confidence intervals were collected for each study and then combined using the random effect (Mantel-Haenszel) method. Summary hazards ratio estimates were provided for each subgroup and full collection of studies. Hazard ratios compared patients with a NLR lower than the cutoff with patients that had a NLR higher than the cutoff value. The NLR cutoff value was unique in each study. Heterogeneity was assessed by the Q and I-squared statistics, calculated for each subgroup and for the full collection of studies. Forest plots were created to show primary and secondary endpoints. Meta regression was created to explore the heterogeneity between OS and NLR cutoff values. Wilcoxon rank sum test evaluated difference of NLR cutoff between subgroups.

## SUPPLEMENTARY MATERIALS FIGURES


