# Supplementary material for: Neutrophil-to-lymphocyte ratio as prognostic indicator in gastrointestinal cancers: a systematic review and meta-analysis
Source: Oncotarget. 2017 Mar 16;8(19):32171–89. doi: 10.18632/oncotarget.16291 (PMC5458276; doi:10.18632/oncotarget.16291)
Supplement: Supplementary file 1 [file oncotarget-08-32171-s001.pdf]

Neutrophil-to-lymphocyte ratio as prognostic indicator in gastrointestinal cancers: a systematic review and meta-analysis

Supplementary Material

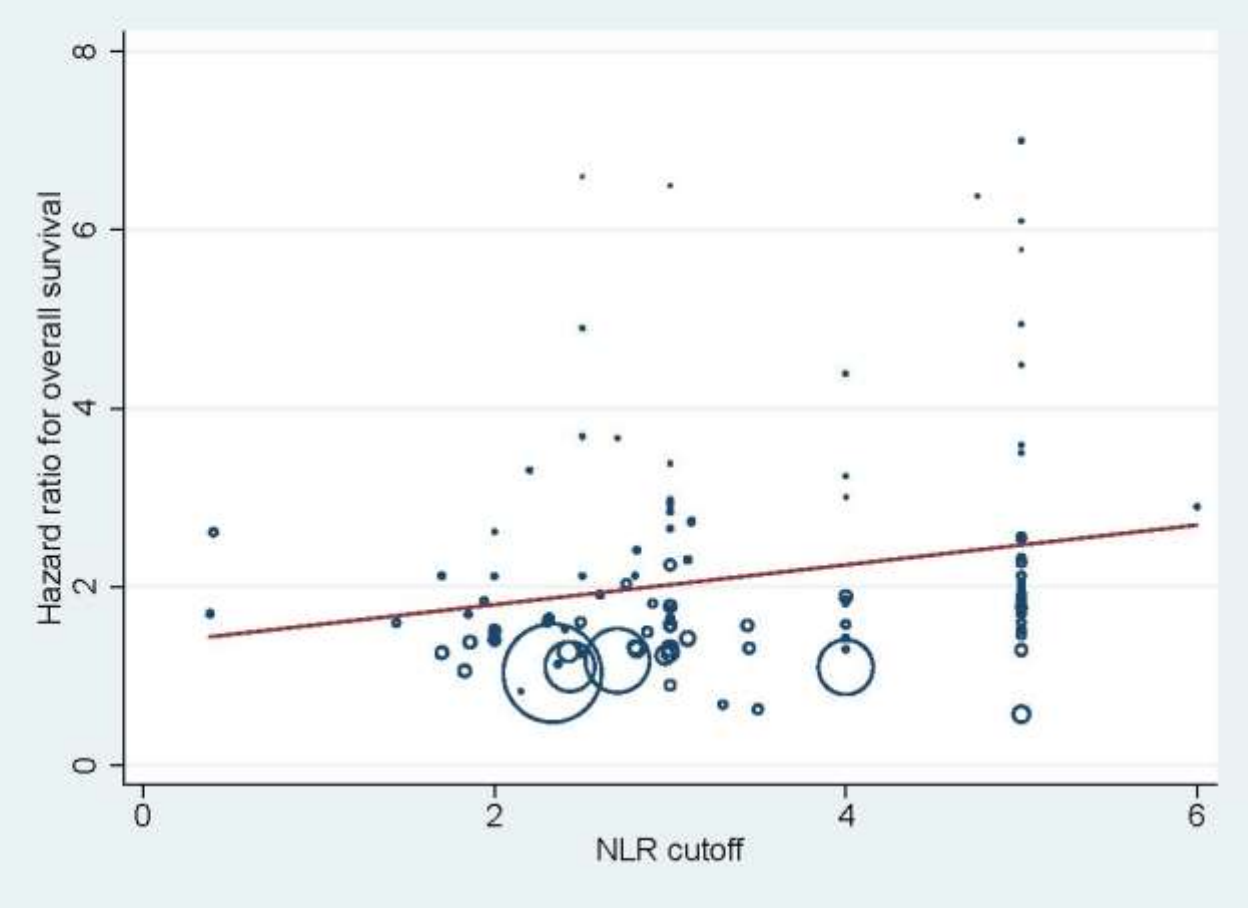

Supplementary Figure 1: Meta-regression of overall survival to neutrophil to lymphocyte ratio cutoff

Figure legend: Evaluate the association between cutoff point of NLR and the HR for OS. Shows minor but statistically significant association between NLR cutoff and the hazard ratio for OS (β=0.224; P=0.019)

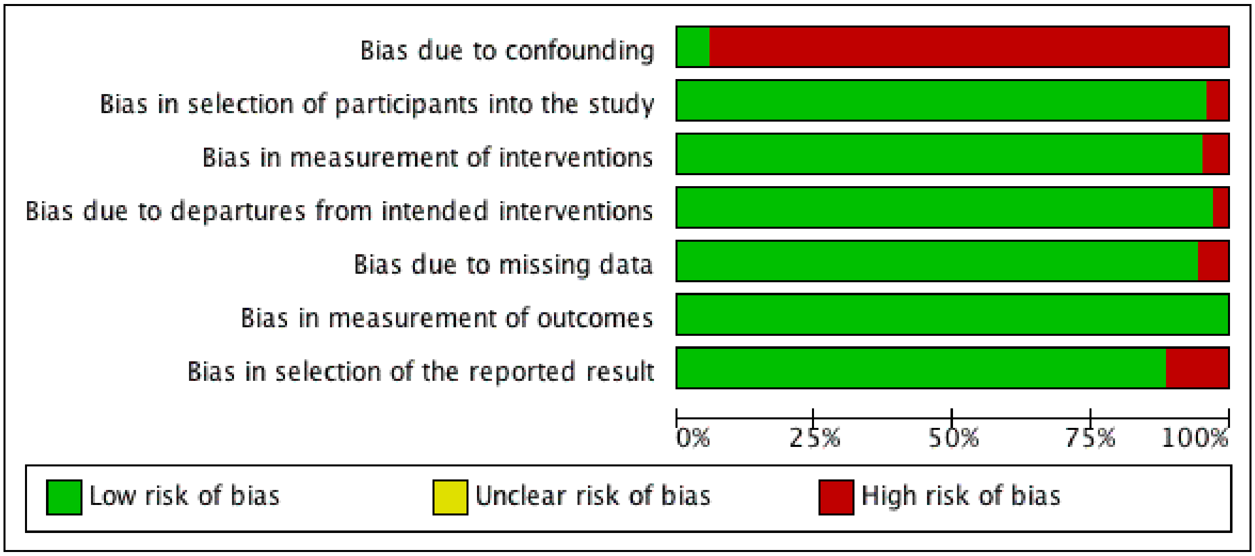

Supplementary Figure 2: Risk of bias summary graph of included studies

Figure legend: Risk of bias graph reflects review authors' judgments about each risk of bias item presented as percentages across all included studies.

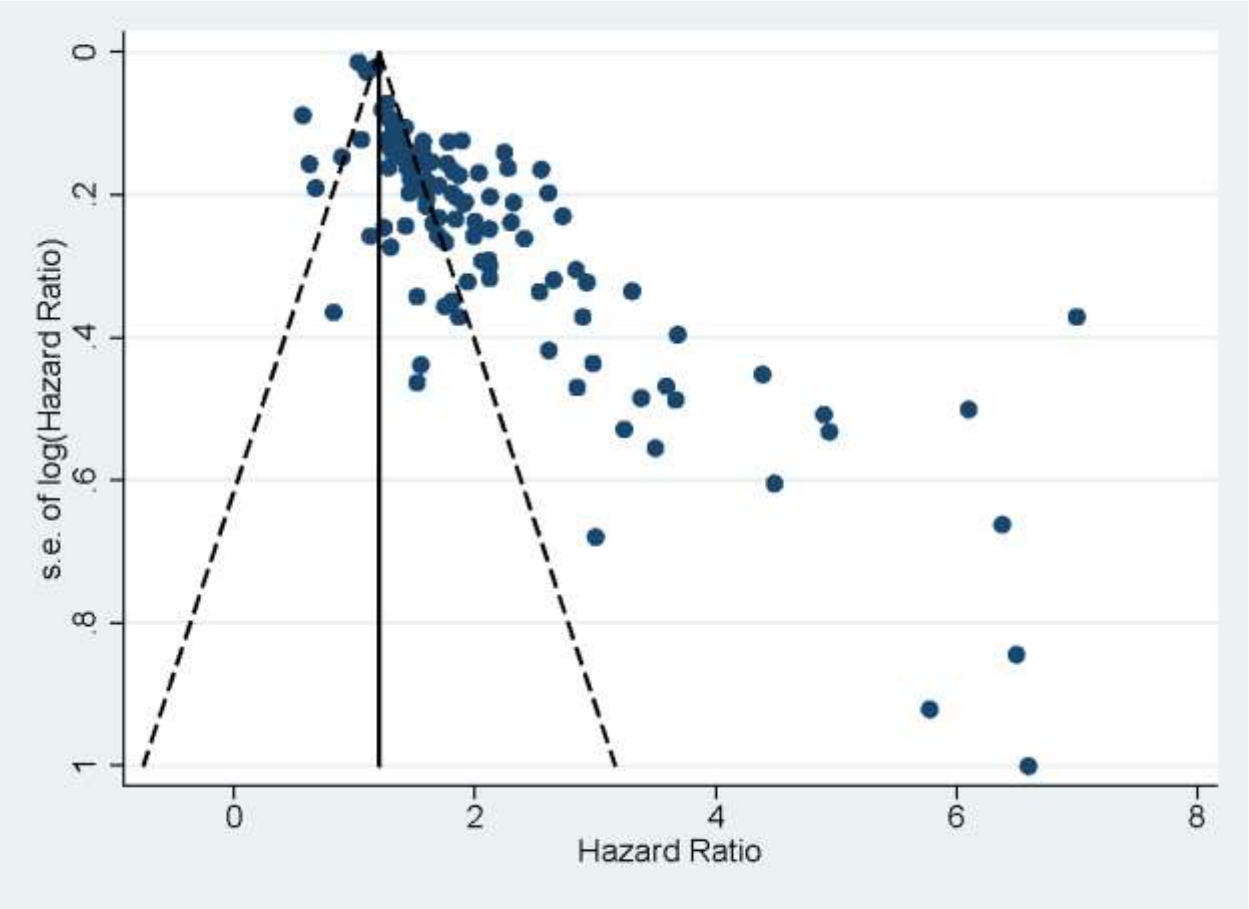

Supplementary Figure 3: Random effect funnel plot analysis.

Figure legend: Random effect funnel plot analysis shows publication bias in the included studies due to skewed distribution.

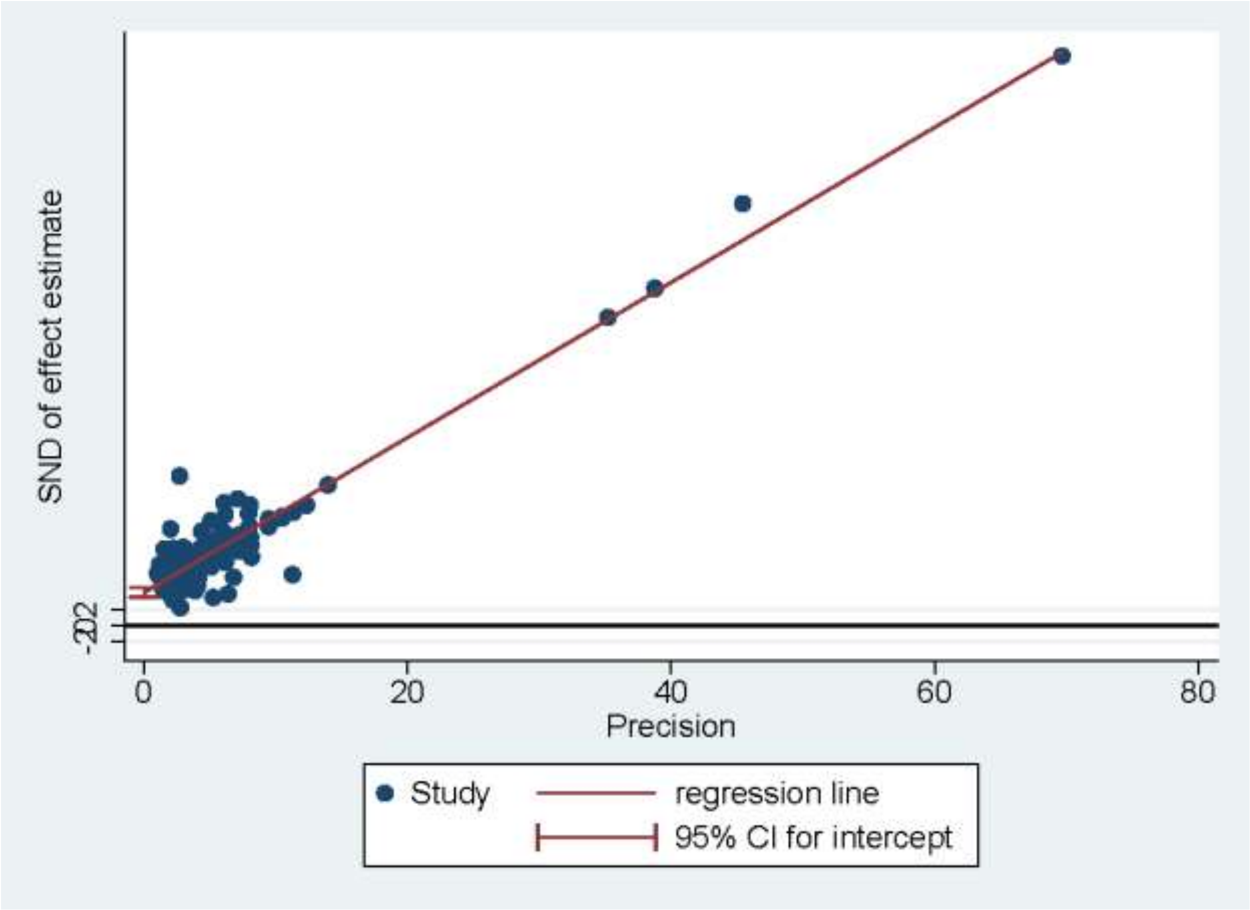

Supplementary Figure 4: Egger's Regression line.

Figure legend: The regression line not originating in the Y-axis zero Indicating publication bias (Bias= 4.165 (95% CI 3.546 to 4.784) P<0.001) as well as a P<0.05 indicating small study effect.

Supplementary Methods. Details of the search strategy, risk of bias, and data extraction for meta-analysis.

Search strategy: Individualized search strategies by (R.C.B.) were utilized to optimize search criteria for each online database.

Medline / PubMed (National Library of Medicine)
Performed: 03/02/2016
((((((neutrophil lymphocyte-ratio) OR NLR) OR neutrophil-lymphocyte ratio) OR neutrophil-lymphocyte-ratio) OR neutrophil lymphocyte ratio))
AND
((cancer OR carcin* OR tumor OR tumour OR neoplasm OR adenocarcin* OR lymphoma OR carcin* OR metasta* OR adenocarcin* OR fibroadenom* OR hepatoma OR hepatoblastom* OR malignan* OR gist OR oncolog* OR rectal OR colorectal OR gastrointestinal OR gastroesophageal OR hepatocellular OR pancreatic OR cholangiocarcinoma OR gastrointestinal stromal tumor OR gist OR anal))
N = 1554

Scopus (scopus.com)
Performed: 03/02/2016
((((((neutrophil lymphocyte-ratio) OR NLR) OR neutrophil-lymphocyte ratio) OR neutrophil-lymphocyte-ratio) OR neutrophil lymphocyte ratio))
AND
((cancer OR carcin OR tumor OR tumour OR neoplasm OR adenocarcin OR lymphoma OR carcin OR metasta OR adenocarcin OR fibroadenom OR hepatoma OR hepatoblastom OR malignan OR gist OR oncolog OR rectal OR colorectal OR gastrointestinal OR gastroesophageal OR hepatocellular OR pancreatic OR cholangiocarcinoma OR gastrointestinal stromal tumor OR gist OR anal))
N = 911

ScienceDirect (Elsevier)
Performed: 03/02/2016
((((((neutrophil lymphocyte-ratio) OR NLR) OR neutrophil-lymphocyte ratio) OR neutrophil-lymphocyte-ratio) OR neutrophil lymphocyte ratio))
AND
((cancer OR carcin* OR tumor OR tumour OR neoplasm OR adenocarcin* OR lymphoma OR carcin* OR metasta* OR adenocarcin* OR fibroadenom* OR hepatoma OR hepatoblastom* OR malignan* OR gist OR oncolog* OR rectal OR colorectal OR gastrointestinal OR gastroesophageal OR hepatocellular OR pancreatic OR cholangiocarcinoma OR gastrointestinal stromal tumor OR gist OR anal))
N = 2076

Embase (embase.com)
Performed: 03/02/2016
'neutrophil'/exp OR neutrophil AND 'lymphocyte ratio' OR nlr OR 'neutrophil lymphocyte' AND ratio OR 'neutrophil lymphocyte ratio'/exp OR 'neutrophil lymphocyte ratio' OR 'neutrophil'/exp OR neutrophil AND ('lymphocyte'/exp OR lymphocyte) AND ratio
AND
('cancer'/exp OR cancer OR 'tumor'/exp OR tumor OR 'tumour'/exp OR tumour OR 'neoplasm'/exp OR neoplasm OR 'lymphoma'/exp OR lymphoma OR carcin* OR metasta* OR adenocarcin* OR fibroadenom* OR 'hepatoma'/exp OR hepatoma OR hepatoblastom* OR malignan* OR gist OR oncolog* OR 'rectal'/exp OR rectal OR colorectal OR gastroesophageal OR hepatocellular OR pancreatic OR 'cholangiocarcinoma'/exp OR cholangiocarcinoma OR gastrointestinal AND stromal AND ('tumor'/exp OR tumor) OR gist OR anal)
N=47

Cochrane Library (Wiley Online Library)
Performed: 04/07/2015
((((((neutrophil lymphocyte-ratio) OR NLR) OR neutrophil-lymphocyte ratio) OR neutrophil-lymphocyte-ratio) OR

neutrophil lymphocyte ratio))
AND
((cancer OR carcin* OR tumor OR tumour OR neoplasm OR adenocarcin* OR lymphoma OR carcin* OR metasta* OR adenocarcin* OR fibroadenom* OR hepatoma OR hepatoblastom* OR malignan* OR gist OR oncolog* OR rectal OR colorectal OR gastrointestinal OR gastroesophageal OR hepatocellular OR pancreatic OR cholangiocarcinoma OR gastrointestinal stromal tumor OR gist OR anal))

N = 6

Risk of Bias Analysis

For risk of bias aggregate analysis we used excel for descriptive analysis. We also grouped risk of bias evaluation into low or high risk of bias (moderate risk and above was classified as high risk) to create bias summary in Cochrane Software (Review Manager (RevMan) [Computer Program] Version 5.3. Copenhagen: The Nordic Cochrane Centre, The Cochrane Collaboration, 2014, available at: http://tech.cochrane.org/revman/download [accessed June 2016]). Funnel plot and regression line also evaluated publication bias.

Data extraction

Pre-made data entry sheets using Microsoft Excel included author, year, country, journal, study type, study size, number male/female, GI cancer site, cancer stage, treatment, NLR, whether c-index was performed followed by OS, DFS, PFS, and CSS.
